# Validity of Accelerometers for the Evaluation of Energy Expenditure in Obese and Overweight Individuals: A Systematic Review

**DOI:** 10.1155/2020/2327017

**Published:** 2020-08-04

**Authors:** Silvia Pisanu, Andrea Deledda, Andrea Loviselli, Inge Huybrechts, Fernanda Velluzzi

**Affiliations:** ^1^Department of Biomedical Sciences, University of Cagliari, Cagliari, Italy; ^2^Department of Medical Sciences and Public Health, University of Cagliari, Cagliari, Italy; ^3^International Agency for Research on Cancer, Nutrition and Metabolism Section, Lyon, France

## Abstract

**Objective:**

Even though the validity of accelerometers for the measurement of energy expenditure (EE) has been demonstrated for normal-weight individuals, the applicability of this instrument in obese individuals remains controversial. This review aims to summarize the level of agreement between accelerometers and the gold standards (indirect calorimetry and doubly labelled water) for the measurement of energy expenditure (EE) in obese or overweight individuals.

**Methods:**

The literature search was limited to comparison studies assessing agreement in EE determination between accelerometers and indirect calorimetry (IC) or doubly labelled water (DLW). We searched in PubMed and in Scopus until March 1, 2019. The analysis was restricted to obese or overweight adult individuals. The following descriptive information was extracted for each study: sample size, characteristics of participants (sex, age, BMI, fat mass percentage, any pathological conditions, modality of recruitment in the study, and exclusion criteria), accelerometer description (model, type and body position), and type of gold standard and validity protocol (duration, conditions, and requirements during and before the experiment). Three review authors independently screened the obtained results, and the quality of the selected articles was assessed by the QUADAS-2 tool.

**Results:**

We obtained seventeen eligible articles, thirteen of which showed concerns for the applicability section, due to the patient selection. Regarding the accelerometers, nine devices were validated in the included studies with the BodyMedia SenseWear® (SWA) being the most frequently validated. Although correlations between accelerometers and the gold standard were high in some studies, agreement between the two methods was low, as shown by the Bland–Altman plots.

**Conclusions:**

Most accelerometer estimations of EE were inaccurate for obese/overweight subjects, and authors advise to improve the accuracy of algorithms for SWA software, or the predicted equations for estimating EE from other accelerometers.

## 1. Background

The assessment of energy expenditure (EE) is essential both in healthy individuals, such as sporty people, and in clinical studies, for the establishment of the amount of physical activity associated with energy balance, fitness, and health benefits [1]. Furthermore, the knowledge of EE plays a central role in the evaluation and management of all conditions that require weight loss or weight maintenance, like obesity [2], and in all the clinical conditions in which physical activity might have a therapeutic value, like hypertension, stroke, coronary heart disease, type I and type II diabetes, metabolic syndrome, and cancer. Given that the fat loss induced by a certain physical exercise has a great individual variability [3], a correct measurement of EE supports a personalized management of weight loss. The physical fitness assessment of overweight and obese patients is fundamental, considering the important benefits of physical exercise as therapy in this population, not only to counteract the cardiovascular risk but also for maintaining the muscle tone, increasing the metabolic rate, and decreasing the risk of common chronic diseases [4].

The traditional gold standard method, the direct calorimetry, has been largely replaced by indirect calorimetry, due to its practicality and cost-effective use. The indirect calorimetry (IC) and the doubly labelled water (DLW) method are the most commonly used gold standards for determining EE [5, 6]. The DLW technique is suitable for use in free-living contexts and provides an accurate measure of total EE (TEE). However, the cost and the requirement of isotope ratio mass spectrometry for analysis prohibit DLW from being used in large population studies. Furthermore, this technique provides an accurate measure of TEE, but no information on physical activity (PA) patterns in terms of frequency, duration, intensity, and energy expenditure is available [6].

IC assesses the amount of heat generated by the oxidation of food substrates, which are converted into CO_2_, H_2_O, and heat. Specifically, EE is calculated by measuring the amount of oxygen used, and carbon dioxide released by the body [5, 7]. The IC allows for real-time measurement of PA, adding the dimensions of duration and intensity. However, the limited access to the equipment and the technical knowledge required for supervision limit the use of portable IC for true field settings or for studies on large populations [8].

The accelerometer represents a valid, noninvasive method for measuring PA under free-living conditions. Accelerometers are designed as small, lightweight, unobtrusive portable devices, with very low operating costs, able to assess PA [9]. Commercial accelerometers usually convert the magnitude of accelerations to provide “activity counts” per defined period of time (epoch). The activity counts represent the estimated intensity of measured activities during each time period. Several regression equations can be derived or validated for different accelerometers to better match the exact EE of physical activities among individuals [10]. Uniaxial accelerometers measure accelerations in one direction, usually in the vertical plane, whereas triaxial accelerometers measure accelerations in the anteroposterior, mediolateral, and vertical direction [11].

The BodyMedia SenseWear® Armband (SWA) is a sleek, wireless, and wearable body monitor that enables continuous physiological monitoring outside the laboratory [12].

The SWA is worn on the posterior side of the master arm and uses a unique combination of sensors. A sensor that detects the heat flow measures the amount of heat dissipated by the body. The skin temperature and the temperature near the instrument are measured by sensitive thermistors. The device also measures the galvanic skin response, which varies with physical and emotional stimuli. An accelerometer follows the movements of the arm and provides information on body position. Individual baseline data, i.e., age, sex, weight, and height, have to be inserted for allowing the activation of the device [12]. Having more sensors is very important for the ability of SWA to accurately monitor physiological conditions. In fact, the presence of multiple sensors allows for the disambiguation of the contexts that could confuse a single sensor. For example, if the movement of a person is high, it may be caused by an exercise or be due to being in a vehicle in movement. However, the variations in temperature, sweat, and heat flow are generally very different for these two situations [9]. The software algorithms use the physiological signals of all sensors to detect the context and then apply the correct formula for the estimation of energy consumption based on sensor values. SWA is able to recognize many basic activities, such as lifting weights, walking, running, cycling, resting, and going by car, bus, or train. Other activities are classified as combinations of these basic activities. The sequential release of software (i.e., version 5.0, 6.1, 7.0, and 8.0) included refined algorithms (i.e., v 2.0, 2.2, and 5.0) designed to improve accuracy and utility [13].

Although the reliability of SWA for the measurement of both REE and EE during physical exercises or in free-living conditions has been demonstrated in different studies considering normal-weight healthy individuals, its applicability remains controversial [14–20]. Under free-living conditions, SWA (software versions 6.1 and 7.0) demonstrated a good agreement with DLW in the Bland–Altman plot and high values of intraclass correlation coefficient (ICC) (>0.80) in healthy subjects [16]. Similarly, laboratory experiments demonstrated reliable estimates of EE (no significant differences versus IC in mean ± SD, or correlations with IC estimates between 0.47 and 0.69) [19, 20]. However, Zorrilla-Revilla et al. found an important overestimation (41.31%) of EE when walking carrying load, while Tucker et al. obtained a significant underestimation (18%) of EE in multiple trials (SWA version 7.0 in both the studies). Regarding its accuracy for the measurement of REE, in the study of Malavolti et al. on healthy subjects, Bland–Altman plot showed no difference in REE determination between SWA (version 4.0) and IC [17], and authors judged the SWA as a reliable device for measuring REE in healthy subjects. In line with these findings, in the study of Zorrilla-Revilla et al., the SWA (version 7.0) estimates of RMR in healthy adults were associated with small error scores (mean absolute percentage error = 17.31%; mean difference = 11.1%) [14].

Overweight and obesity are responsible for biomechanical modifications during walking, with loss of efficiency [21]. As a consequence of the lack of efficiency, accelerometers may overestimate EE of obese individuals due to excessive body motion (greater body movement associated with reduced mechanical efficiency) [19, 37]. Other concerns on the applicability of accelerometers in obese individuals are the obvious differences in the placement of these devices with respect to the center of mass of the body, and the different patterns of PA in daily life. Consequently, the accelerometer output, the EE estimates that derive from it, and the accuracy of these estimates compared with criterion methods can be affected [9]. An advantage of SWA can be the potential ability to detect false motion and the detection of nonambulatory physical activity, which is provided by the combination of the accelerometer data with the other physiological sensors. However, it is possible that higher levels of body fatness may impact the accuracy of the existing algorithms [37].

The aim of our systematic review is to summarize the existing evidences for the level of agreement between accelerometers and gold standards (IC or DLW) for the measurement of EE in obese or overweight individuals.

## 2. Methods

Cohort studies, intervention studies, and validation studies were considered in our analysis. In order to be included, studies were required to report the comparison between accelerometers and gold standard results in the same individuals and in the same conditions. Our analysis was restricted to obese or overweight adult individuals (older than 18 years, BMI ≥ 25 kg/m^2^). Studies on participants with illnesses or conditions that may affect EE (fever, infections, immunodeficiency syndrome, cancer, and traumatic injury) or undergoing any type of elective surgical procedures were excluded. The search was limited to publications written in English.

### 2.1. Search Method

The literature search was limited to method comparison studies assessing agreement in EE determination between accelerometers and IC or DLW, which we considered as gold standards [22, 23]. The choice of these methods was based on their common use in clinical practice and in intervention and validity studies. We searched in PubMed and in Scopus (final search on 1 March 2019), using combinations of the following keywords: “accelerometer,” “SenseWear armband,” “accelerometry,” “motion sensor,” “activity monitor,” “armband,” “multi-sensor,” “obese,” “overweight,” “obesity,” “validation study,” “indirect calorimetry,” “double labelled water,” “doubly labelled water.” Our search strategy was designed to incorporate studies that included the use of both the accelerometers device and the gold standard in the same population. Three review authors independently screened the obtained results. In order to increase the sample size, each study selected for being included in the review was inserted in Google Scholar, using the function “cited by” and “correlated.” The reference lists of included studies were also checked for additional relevant studies.

The eligibility of each study was initially based upon details presented in the abstract followed by reading the full text of all possible studies. Disagreements on the inclusion or exclusion of each study were resolved by consensus.

### 2.2. Data Extraction

Data relating to sample size, characteristics of individuals (age, sex, BMI, fat mass percentage, and any pathological conditions), exclusion criteria, accelerometers (model and location, and software or algorithm used for data analysis), gold standard method, protocol of the experiment, primary results of outcome measures, and reported statistics including statistical significance and conclusion results were extracted. Two review authors extracted the data listed above from the included studies and the third author checked the extracted data.

### 2.3. Study Selection Process

Our initial search yielded 343 results. After the removal of duplicates, we obtained a total of 273 articles.

The titles and/or abstracts were screened by three authors. A total of 33 publications were identified as potentially relevant according to inclusion criteria. We excluded 24 full-text articles for the following reasons: in one study, there were no data on the accelerometer; in three studies, accelerometer and gold standard were used in different conditions; in one study, the EE from accelerometer was not calculated; in twelve studies, the accelerometer was not compared with a gold standard for the measurement of EE; in three studies, the individuals were of normal weight; in one study with a population of different BMI, this index was not considered in the results; in two studies, the mean difference between accelerometer and gold standard was not reported; and one study enrolled lactating women. Consequently, we obtained a total of 9 eligible articles. After the search performed in Google Scholar and the screening of the references of eligible studies, 8 additional articles were selected, obtaining a total of 17 included studies.

### 2.4. Quality Assessment

According to Whiting et al. [24], we evaluated the quality of the studies using the second version of the Quality Assessment of Diagnostic Accuracy Studies (QUADAS-2) checklist. This tool consists of four key domains: patient selection, index test, reference standard, and flow of patients through the study and timing of the index tests and reference standard (“flow and timing”). Each domain is assessed in terms of risk of bias and concerns regarding applicability. Risk of bias can be judged as “low,” “high,” or “unclear” based on the provided signalling questions. If all the questions for a domain are answered “yes,” the risk of bias is judged as “low,” while if any question is answered “no” the risk of bias is taken into consideration by the reviewers. Concerns regarding applicability are rated as “low,” “high,” or unclear. Unclear category is used if insufficient data were reported to permit a judgement.

## 3. Results

### 3.1. Characteristics of Participants in the Selected Studies

Table 1 shows the characteristics of the participants enrolled in the selected studies. The sample size ranged from 10 to 264 and the mean age ranged from 25 to 82 years (43 ± 13 years). BMI ranged from 29.2 to 43.2 kg/m^2^ (33.4 ± 6.7 kg/m^2^). In 8 studies, all the participants were obese or overweight [9, 25, 30–32, 37, 39, 40]. In the study of Elbelt et al. [29], the population was divided into 4 groups according to BMI, the first including both normal-weight and overweight individuals. Similarly, Swartz et al. divided the sample into three groups based on the BMI category (normal weight, overweight, and obese) [37].

In the study of Correa et al., participants were recruited from two clinical trials aimed to weight loss. The first trial enrolled only participants whose BMI was >25 and <40, while the second trial enrolled participants whose BMI was >18.5 and <40 [27].

Five studies recruited healthy volunteers whose mean BMI was indicative of overweight/obese status [26, 28, 33, 35, 38].

In two studies, the population included patients with nonalcoholic steatohepatitis [29, 30]. In addition, in the study of Machač et al., participants were volunteers with type I or type II diabetes, being obese as a group, as indicated by the mean BMI [34].

Table 2 shows the characteristics of the included studies and the validity results.

### 3.2. Outcome Measures

The validity of accelerometers for the measurement of REE was evaluated in 5 studies [25, 29, 30, 36, 40]. Nine studies investigated the validity of accelerometers for the measurement of PAEE, considering the execution of specific physical activities in laboratory conditions [9, 26, 28, 31, 33, 34, 37, 38], and 5 studies validated the accelerometers for the measurement of TEE or PAEE under free-living conditions [9, 27, 32, 35, 39]. All the studies used a range of statistics to assess agreement between the accelerometer and the chosen reference test. The majority of the included studies used Bland–Altman plots and Pearson correlation coefficients to show agreement and association between accelerometer and gold standard, respectively. The mean difference between accelerometer and gold standard was inserted in almost all the included studies, expressed as an absolute value or as a percentage, but the significance of the difference was not always specified. The standard deviation of the mean difference was also not always reported.

### 3.3. Reference Methods

For REE and PAEE, IC was used as the gold standard in all the included studies. In total, 3 and 5 different indirect calorimetry devices were employed for REE and PAEE, respectively. For the measurement of TEE or PAEE in free-living conditions, DLW was used as the gold standard in all the included studies.

### 3.4. Validity Protocol

Regarding accelerometers, 9 devices were validated in the included studies: SWA was the most validated, being evaluated in 11 studies using different software versions (from 4.0 to the most recent 8.1) [25–27, 29–31, 34–36, 38, 40]. The uniaxial Caltrac accelerometer was used in one study [32]; the RT3 triaxial accelerometer was used in one study, compared with the triaxial TriTrac-R3D [9]; one study evaluated the accuracy of the Fitbit Charge 3-axis accelerometer [28]; one study validated the Kenz Lifecorder EX accelerometer [37]; the ActiGraph GT3X + triaxial accelerometer was validated in one study [33]; one study tested the validity of the Actical omnidirectional accelerometer [27] and one study used the TracmorD triaxial accelerometer [39].

EE was assessed in laboratory during different exercise tests [9, 26, 28, 31, 33, 34, 36, 37], with IC and accelerometer at the same time. In the study of Thorp and colleagues, the accuracy of the accelerometer in estimating EE was evaluated in order to determine whether alternating bouts of sitting and standing at work influenced daily workplace EE [38]. In 4 studies, the exercise tests were performed after a fasting period of at least 2 hours [26, 31, 37, 38], and in 2 studies, abstention from physical activities for at least 3 hours before the test was required [31, 38].

### 3.5. Risk of Bias and Study Methodology Quality Assessment Scores

Risk of bias was low in the patient selection. However, in 8 studies [9, 25, 28, 33,34, 36, 37, 40], it was judged as high for index test and gold standard items, due to the absence of an at least 2 hours of fasting period and/or an at least 15 minutes of resting period before the test. In 5 studies, the risk of bias was judged as high for the lack of individuals included in the final analysis, due to technical problems with the accelerometers [9, 29, 38], or because they did not finish the accelerometer assessment or the DLW dosing period [27, 35]. Referring to concerns with the applicability of the proposed test, the risk was judged as low for index test and gold standard, but the majority of studies presented an issue in the patient selection [9, 25, 27–30, 33–39] due to not having excluded smoking individuals and/or individuals taking medication that could affect EE.

### 3.6. Validity Results

#### 3.6.1. REE

One study obtained an underestimation of REE_SWA_ although the statistical significance was not specified [36]. However, a significant overestimation of SWA was observed in all the other included studies [25, 30, 37, 40]. Four studies showed results of Bland–Altman plots [25, 30, 36, 40]. Pearson's correlation coefficient was reported in three studies [29, 36, 40], in which it ranged between 0.58 (obtained in women) and 0.88 (obtained in the whole population).

In the studies of Bertoli et al. and Elbet et al., Bland–Altman plots showed a low agreement [25, 30]. On the other hand, Papazoglou et al. reported a mean underestimation with narrower limits of agreements [36].

Results of Bland–Altman analysis revealed the tendency of the bias to increase as the REE increased across participants. Authors did not find any relationship between the bias and age, BMI, fat-free mass, total body water, and extracellular water of individuals [36], in agreement with the work of Elbelt et al., in which the bias was not significantly associated with changes in body weight [29]. In the study of Waldburger and colleagues, Bland–Altman plots indicated that SWA systematically overestimated REE in women displaying low REE values and underestimated REE in women displaying high REE values [40].

In 2012, Elbelt et al. proposed an alternative method for the evaluation of REE by SWA by measuring sleep EE (SEE) for 3 consecutive days [30]. Despite the high correlation between the two methods, the mean difference was significant, with around 6% of the included participants being outside the limits of agreements (LOA) (LOA: −715 to −318 kJ/day for the early uninterrupted phase of sleep and −761 to −377 kJ/day for the late uninterrupted phase of sleep) [30].

### 3.7. EE during Different Physical Exercises or Sedentary Behaviours

Five included studies presented Bland–Altman plots [9,26,31,36,38], while Pearson's correlation coefficients were indicated only in three studies [31, 34, 38]. A general trend toward overestimation can be noticed (see Supplementary Material 1). However, the study protocol differs greatly among the included studies.

In the study of Papazoglou et al., the validity of SWA for the estimation of PAEE was evaluated using Inner View Research Software 4.0. for 3 different physical activities (pedalling on an ergometer, stepping, and walking on a treadmill). The overestimation of SWA was significant, and the Bland–Altman plots showed no agreement for all the 3 physical activities [36].

A more recent version of SWA (Inner View Research Software 6.1) was used in the study of Erdogan et al. The considered exercise consisted of rowing for 10 minutes at two different intensities: authors obtained a good agreement between SWA and IC for high-intensity exercise, but the overestimation of SWA was significant when rowing at moderate intensity [31]. Correlation coefficients indicated a significant association with the gold standard for both the intensities.

Lastly, in the study of Bhammar et al., the SWA's versions 7.0 and 8.1 were validated for the measurements of EE during a structured and a semistructured routine, including a range of activities from light to vigorous intensity. In the structured routine, both the versions of SWA provided a significant overestimation of EE. On the other hand, in the semistructured routine, the most updated version of the software provided estimates not significantly different than the gold standard, as confirmed by the narrow limits of agreements in the Bland–Altman plot.

In the study of Jacobi et al., two different experiments were performed. In experiment 2, the validity of a triaxial accelerometer (RT3) and the validity of TriTrac-R3D (in which 3 accelerometers are incorporated) were compared with IC for walking at 3 different speeds. In addition, the validity of the same accelerometers (RT3 and TriTrac-R3D) in estimating PAEE was evaluated against DLW in a group of 13 overweight or obese women in free-living conditions (experiment 1, see Table 1 and Table 2) [9]. Regarding experiment 2, both devices showed a trend toward overestimation, but RT3 measures were more accurate. Despite the better results obtained by RT3, authors specified that this accelerometer presents some limitations when the individual level is considered, as shown by Bland–Altman plots [9].

Dondzila and Garner evaluated the accuracy of the consumer-grade accelerometer Fitbit Charge 3-axis during walking and jogging: an important underestimation was obtained for both the activities, suggesting a low reliability of the device [28].

In the study of Imboden et al., the validity of the research-grade accelerometer ActiGraph GT3X was tested in a semistructured routine, including both sedentary and ambulatory/cycling activities. A large underestimation of PAEE was observed, mostly driven by the household activities included in the routine [33].

Machač et al. enrolled adult volunteers with type I or type II diabetes, in order to verify the accuracy of accelerometers in this specific population. The mean BMI of the participants indicated that they were, as a group, obese. The protocol consisted of 3 sessions (15 minutes each) of walking on a treadmill. The SWA (software version 6.1) provided accurate estimates at different speeds (as demonstrated by Pearson's correlations between 0.63 and 0.79). Based on the authors' interpretation, the positive results were due to the reasonable duration of the protocol, considering that shorter experiments are more prone to bias [34].

In the study of Swartz et al., participants were divided into 3 categories based on BMI (normal weight, overweight, and obese), and results were presented for all the samples and by BMI group. The accelerometer Kenz Lifecorder EX was validated during a 6-stage walking protocol on a treadmill. In overweight and obese participants, there was a trend toward overestimation, and in general, the authors concluded that the instrument was not accurate for measuring EE in individuals with varying BMI [37].

The study of Thorp et al. was the only one to consider sedentary behaviour, defined as any waking behaviour characterized by an EE of 1.5 or fewer METs while sitting, reclining, or lying and including most office work, driving a car, standing quietly, and sitting while watching television [38]. In this study, the SWA software version 7.0 was evaluated. Bland–Altman plots showed a moderate agreement between the two methods when sitting, and a strong agreement when standing, while correlation coefficients indicated a significant association with the gold standard for both standing and sitting.

### 3.8. TEE and PAEE under Free-Living Conditions

Five included studies performed Bland–Altman analysis for comparing EE measured by IC and accelometers [9, 27, 32, 35, 41].

In the study of Fogelholm et al., the accuracy of the Caltrac uniaxial accelerometer in the measurement of TEE was evaluated: even if the accuracy of the instrument was good at a group level, at individual level differences were large [32].

An underestimation of EE in free-living conditions was obtained by the work of Jacobi et al. RT3 limits of agreement were smaller than TriTrac-R3D, but presented limitations at individual levels [9].

Correa et al. selected a subsample of participants from 2 clinical trials aimed to weight loss and evaluated the validity of 3 different accelerometers for the measurement of AEE and TEE under free-living conditions. In contrast with other studies included in the review [9, 32], two accelerometers provided accurate estimates. More specifically, Bland–Altman plots showed that SWA and IDEEA accurately estimated TEE, and the IDEEA accurately measured AEE. On the other hand, the performance of Actical was low. Authors stated that the study provides a modest support for the assertion that multisensor activity monitors produce more accurate estimates of AEE and TEE, compared with traditional accelerometers; however, they also expressed the need for further validation research [27].

In line with the findings of Correa et al., the study of Mackey et al. tested the accuracy of TEE and AEE estimates of the SWA, using software versions 6.1 and 5.1 in a sample of older participants (78–89 years old), which were overweight as a group. Both versions showed high Pearson's correlation coefficients (*R* > 0.75) for TEE. On the other hand, AEE was underestimated by both versions 6.1 and 5.1. Nevertheless, Bland–Altman plots revealed no systematic bias when considering both TEE and AEE [35].

Finally, Valenti et al. enrolled obese and overweight individuals in order to validate a new specific equation for the estimation of TEE from TracmorD accelerometer under free-living conditions. The developed equation allowed valid assessment of physical activity level (PAL, calculated as TEE/sleeping metabolic rate) and AEE/body weight (AEEkg). More specifically, PAL estimates were highly correlated with the gold standard measurements (*R* = 0.69), and the errors were correlated with PAL but not with BMI. Similarly, AEEkg and predictions from the new equation were highly correlated (*R* = 0.76, *p* < 0.001) and the errors did not correlate with the BMI [39].

## 4. Discussion

Despite the numerous studies aimed to validate the use of accelerometers in estimating EE, there are no systematic reviews that focus on the validity of these devices in overweight and/or obese individuals.

The use of accelerometers in this population presents some potential issues. For instance, for the same physical activity effort, obese/overweight subjects spend more energy than normal-weight individuals [42], due to the increased fatness. In fact, the physiological energy expenditure is influenced by both body weight and movement efficiency and so may not necessarily reflect the intensity and amount of body movement [43]. In addition, the accuracy of accelerometers is reduced if the sensor is positioned at an angle, which may happen more often in overweight or obese individuals [43, 44], due to the increased fat mass. Moreover, it has been demonstrated that the error of accelerometers' estimates is affected by the activity type and intensity (being higher in the case of vigorous activities and sedentary behaviors) and by differences in body weight, with increasing BMI being associated with increased bias [45, 46]. Furthermore, some accelerometers, such as SWA, require the insertion of subjects' characteristics (gender, sex, and smoking status) in the equations, including anthropometric parameters, whose measurement itself can represent a source of bias, especially for the estimation of REE [14].

In our review, accelerometers' estimations of EE in obese and overweight individuals are shown to be inaccurate in many studies and most authors advise to improve the accuracy of algorithms for the SWA software or the predicted equations for the other accelerometers.

Even though results on the validity of accelerometers in obese and overweight individuals remain contradictory, it is possible to notice a trend toward overestimation for REE (see Supplementary Material 1), which was measured by SWA in the included studies.

The findings on REE obtained in our review are in contrast with those obtained in the normal-weight population. In the study of Malavolti et al., SWA (software 4.0) provided accurate estimates of the REE, not significantly different than those of the IC, as confirmed by the Bland–Altman plot [17]. Similarly, Casiraghi et al. obtained a high Pearson's correlation (*R* = 0.95) when testing the accuracy of SWA (software 6.1) for measuring REE in healthy normal-weight individuals [47]. In addition, Zorrilla-Revilla et al. found small mean absolute percentage error (MAPE) and percentage mean change in healthy adults, when measuring RMR using the SWA mini (software 7.0) [14].

In our review, a similar trend toward overestimation was obtained also for PAEE, compared with IC (see Supplementary Material 1).

The triaxial accelerometer RT3 seemed to be more accurate than the uniaxial accelerometers for predicting EE in obese and overweight individuals during walking on a treadmill [9]. This is in line with results obtained in the general population, which show an accurate measure of PAEE by the RT3, both under laboratory and the free-living conditions [48, 49].

A previous version of SWA (4.0) was assessed by Papazoglou et al. in obese individuals, during an activity protocol including pedalling, stepping, and walking. Authors recommended to improve the accuracy of the software, though the study was published in 2006 and an old version has been used.

Two of the included studies came to an opposite conclusion when evaluating the accuracy of the more recent software version 6.1 [31, 34]. On the one hand, Erdogan et al. claimed the need to improve the accuracy of the SWA algorithms for obese individuals, and on the other hand, Machač et al. obtained a good accuracy in obese volunteers with diabetes. Regarding the corresponding findings in healthy adults, SWA 6.1 outperformed other activity monitors for the estimation of EE during light- to moderate-intensity semistructured activities [50].

Swartz et al. validated the Kenz Lifecorder accelerometer in volunteers with various BMI (normal weight, overweight, and obese): the accelerometer was judged as inaccurate due to the important overestimation. This result is in line with evidences obtained in normal-weight adults, which show an overestimation of the Kenz Lifecorder at some walking speeds [51].

In one of the included studies, the recent software version of SWA (8.1) showed a better performance than SWA 7.1 in a semistructured routine, which reflected until a certain level the free-living conditions [26]. In agreement with this finding, in a study in which participants were of normal-weight and completed a series of physical exercises, the SWA version 8.1 outperformed the previous one, showing a MAPE of 20% in total [50].

In the included study of Thorp et al. on sedentary behaviours, SWA 8.1 showed a good accuracy when standing, but not when sitting [38]. However, also a study enrolling normal-weight participants obtained an underestimation of SWA during office work (standing or sitting) [53].

In one of the included studies, Imboden et al. found an important underestimation of PAEE during a semistructured routine by the accelerometer ActiGraph GT3X. The same accelerometer proved to be a good tool to predict EE in normal-weight adults during walking, compared with IC, and under free-living conditions, compared with DLW [54, 55]. Interestingly, in the study of Imboden et al., the household activities presented the highest bias [33].

The Fitbit Charge was considered not reliable for the measurement of EE in a walking protocol [28]. In line with this finding, the Fitbit Charge showed to overestimate moderate to vigorous physical activity in normal-weight participants, compared with other research-grade accelerometers [56].

Regarding the measurement of TEE in free-living conditions, two studies obtained important differences at the individual level [9, 32]. It should be specified that in these two studies, only overweight/obese participants were enrolled. The findings related to this population were in contrast with those in healthy subjects [16].

On the other hand, other studies highlighted the potential of the multisensor accelerometers (SWA and IDEEA), compared with traditional accelerometers, finding a good level of agreement with the DLW [27, 35]. However, differences were still large at the individual level.

In the study of Valenti et al., equations developed by the authors specifically for the obese population improved the accuracy of EE predictions, when using the accelerometer TracmorD [39].

We must note two limitations inherent in this systematic review. Firstly, our search method and inclusion criteria have restricted the number of included studies. Secondly, the selected studies varied greatly in population characteristics, accelerometer models and algorithms, and protocols, making it difficult to compare the obtained results. Due to the heterogeneity of the included studies, a meta-analysis was not possible. In addition, the indirect calorimetry is currently considered the most accurate approach for estimating EE in obese individuals and the only accurate approach in extreme obese patients (class III) [5]. On the other hand, DLW is the most accurate and objective measurement for assessing PA in free-living individuals [57], but a greater underestimation of EE has been shown in heavier and fatter subjects [58]. Although the possible underestimation in this specific population should be taken into account, it still remains more accurate than other methods of PA assessment in free-living conditions [57].

For the measurement of REE and PAEE, the different protocols followed in the included works (i.e., hours of fasting and hours of abstention from alcohol and physical activity before the test and duration of the test) reflect the need to publish a standardized protocol for validation studies, with the aim to facilitate the comparison of results. In most of the included studies, exclusion criteria were based on the presence of disease conditions (such as chronic organ disease, cardiovascular disease, and cancer) or medical conditions that could interfere with the execution of the physical exercises. We believe that, in addition, the use of medications known for influencing the thermoregulatory process (e.g., sibutramine, anticholinergics, psychotrops) or that may interfere with energy expenditure (beta-adrenergic and corticosteroids) should be always taken into consideration [31]. We also recommend an abstention from food and caffeine (minimal 4 hours) and moderate-vigorous physical activity (minimal 2 hours). In any case, even when the abstention from physical exercise is not indicated in the protocol, laboratory experiments should be initiated after a period of rest (10–20 minutes) in order to minimize possible effects of recent physical activities such as dressing, driving, or walking [59, 60]. Furthermore, taking into account the well-known effects of smoking in affecting EE [61, 62] and that smoking can affect the measurement of RMR in obese patients [63], smokers should be excluded from the sample population, or the results should be presented considering the smoking status. This exclusion could be avoided in the case of accelerometers that take into account the smoking status in the equations (i.e., SWA) [34].

Once enough studies specific for obese and overweight individuals with standardized protocols will be available, the establishment of correction factors for accelerometer estimations will be possible. It is important to specify that, even if accelerometers do not allow us to correctly evaluate the exact energy balance in overweight or obese individuals, they can be very useful in clinical practice, as observed for supervised physical activity, for the monitoring of behavioural changes and in the consequent motivational stimulus to undertake more active lifestyles.

## Figures and Tables

**Table 1:** Characteristics of participants in the included studies.

Reference	Sample size	Age (SD)	Sex	BMI (SD)	%BF or fat mass expressed in kg (FM)	Characteristics of the population
[25]	169	F = 44 (12); M = 44 (11)	F/M = 127/42	F = 29.8 (5.7); M = 31.2 (4.4)	—	Overweight patients (individuals with acute and chronic organ diseases were excluded)
[26]	34	30.1 (8.7)	F/M = 26/8	26.2 (5.1)	%BF = 30.6 (10.2)	Volunteer adults (all participants were nonsmokers and were not taking any medications for hypertension, diabetes, heart disease, or hyperlipidemia)
[27]	87	42 (13)	F/M = 72/15	31.6 (4.5)	—	87 participants recruited from 2 clinical trials (5 excluded because they did not finish baseline accelerometry assessment, 5 excluded due to BMIs < 25, 7 excluded because they did not successfully complete all aspects of the DLW dosing period; final sample size = 70). General exclusion criteria: pregnant or planning to become pregnant during the trial (females only); previous diagnosis of diabetes, cardiovascular disease, or cancer; use of medications that influence appetite or body weight during the previous 3 months; weight instability
[28]	19	24.6 (3.1)	F/M = 14/5	28.0 (3.8)	—	Volunteer adults having no cardiovascular, respiratory, metabolic, or musculoskeletal disorders with no limitations to exercise, and an age range of 18–30 years
[29]	78	46 (12)	F/M = 55/23	28 individuals of normal weight or overweight ((27.0 (2.0)), 13 individuals with obesity I (32.5 (1.3)), 13 individuals with obesity II (37.7 (2.0)), and 24 individuals with obesity III (48.2 (5.3))	—	Patients about undergoing bariatric surgery or with nonalcoholic steatohepatitis (of which 19 individuals with DM2, 16 individuals with impaired glucose tolerance, and individuals suffering from diseases preventing them to perform normal daily physical activities were excluded)
[30]	81	46 (13)	F/M = 58/23	36.4 (9.3); M = 43.9 (6.5)	—	Outpatients with obesity or nonalcoholic steatohepatitis
[31]	43	34.9 (5.5)	F/M = 27/6	31.2 (3.7)	%BF = 38.3 (5.6)	Obese patients participating in an on-going weight loss program (individuals with a medical condition that could prevent safe participation in maximal exercise testing, or with a medical condition that would require medical clearance or diabetics and patients on medication that might have affected the SWA or the thermoregulatory process were excluded)
[32]	20	40 (4)	F = 20	29.2 (3.0)	%BF = 34.9 (4.7)	Premenopausal volunteer women, no taking any medication or oral contraceptives, and no smoking, pregnant, or lactating
[33]	30	49.2 (19.2)	F/M = 15/15	26.4	—	Healthy volunteers
[9]	10	41.5 (10.9)	F/M = 6/4	34.3 (5.0)	—	Healthy patients, no taking medications known to modify EE
[9]	13	38.3 (10.5)	F = 13	34.2 (6.4)	—	
[34]	19	F = 51.1 (11.0); M = 60.3 (3.1)	F/M = 13/6	31.5 (3.6)	%BF = 35.7 (8.3)	Volunteers with type 1 and type 2 diabetes mellitus (individuals with glycosylated hemoglobin over 7.5%; serious retinopathy, macular edema; serious nephropathy, in the proteinuria stage or renal failure; serious neuropathy of the lower limbs, diabetes leg syndrome; repeated unrecognized hypoglycemia, labile diabetes; another acute disease; or other diseases influencing or increasing risks of physical stress were excluded)
[35]	19	82.0 (3.3)	F/M = 8/11	28.1 (3.8)	—	Sample of participants enrolled in the prospective Health, Aging, and Body Composition Study (started in 1997–1998) that were inserted also in the energy expenditure substudy
[37]	142	46.9 (14.2)	F/M = 105/37	42.3 (7.0)	—	Obese patients and lean and overweight volunteers (individuals with a medical condition that could prevent safe participation in moderate-intensity exercise or that require clinical clearance before participation or with diabetes or taking medication that could affect thermoregulatory process were excluded)
[36]	29	31.2 (3.2)	F/M = 14/15	43.2 (5.3)	—	
[37]	48	33.0 (10.7)	F/M = 28/20	25 individuals of normal weight ((21.6 (2.0)), 12 overweight individuals (27.3 (1.0)), and 11 individuals with obesity (33.2 (2.1))	—	Volunteer adults free from diseases, disorders, or orthopedic conditions that may impair the participants' ability to walk on a motorized treadmill
[38]	23	48.2 (7.9)	F/M = 6/17	29.6 (4.0)	—	Volunteer adults
[39]	36	41 (7)	F/M = 25/11	31.0 (2.5)	—	Overweight and obese volunteers
[40]	264	44.7 (12.5)	F/M = 188/76	F = 41.4 (6.1); M = 43.9 (6.5)	%BF: F = 47.9 (4.5); M = 36.9 (5.2)	Obese patients

%BF: body fat percentage; BMI: body mass index; DLW: doubly labelled water; DM2: diabetes mellitus type 2; SWA: SenseWear Armband.

**Table 2:** Characteristics of the included study and validity results.

Reference	Activity monitor/location	Gold standard	Variables analysed	Protocol	Validity results	Conclusion
*REE*						

[25]	SWA (Inner View® Research Software 4.0)/triceps muscle	IC, using Sensormedics Vmax-29N	REE	At least 30 min of IC + SWA (data collected during the first 5–10 min excluded for allowing the acclimatisation, experiment conducted at thermos neutral environment and in the absence of external stimuli)	(i) *Women*: (1) Mean difference: 230.1 ± 690.4 kJ·d^−1^ (*p* < 0.001) (2) Bland–Altman: LOA (1125; 1582 kJ·d^−1^) (3) Pitman's test: *R* = 0.034 (*p*=0.707) (4) Lin's CCC: (95% CI) 0.579 (0.467; 0.691) (ii) *Men*: (1) Mean difference: 451.9 ± 937.2 kJ·d^−1^ (*p* < 0.001) (2) Bland–Altman: LOA (1381; 2280 kJ·d^−1^) (3) Pitman's test: *R* = −0.221 (*p*=0.180) (4) Lin's CCC*:* (95% CI) 0.583 (0.397; 0.768)	Poor agreement between SWA and IC for the assessment of REE

[29]	SWA (Inner View® Research Software 6.1)/right upper arm over the triceps muscle)	IC, using Deltatrac II	REE	At least 30 min of IC + SWA (data collected during the first 5–10 min excluded for allowing the acclimatisation, experiment conducted under standardized condition, after an overnight fast and after a resting period of at least 30 min)	Data available for 63 subjects Mean difference: 870.2 ± 991.6 kJ·d^−1^ (*p* < 0.001) Pearson's correlation: *R* = 0.826 (*p* < 0.001)	The overestimation of REE by SWA can be a limitation in physiological studies

[30]	SWA (Inner View® Research Software 6.1)/right upper arm over the triceps muscle	IC, using Deltatrac II	REE_IC_ and SEE_SWA_	*REE*_***IC***_: experiment conducted under standardized conditions, after an overnight fast and after a resting period of at least 30 min, with the request not to smoke prior the experiment *SEE*_***SWA***_: EE continuously measured for 3 d. SEE_ep_ and SEE_lp_ data were analysed if the duration was at least 35 min, with the exclusion of the first and the last 5 min	(i) *SEE*_*ep*_: (1) Mean difference: 514.6 ± 895.4 kJ·d^−1^ (*p* < 0.001) (2) Linear regression: *R*^2^ = 0.705 (*p* < 0.001) (3) Bland–Altman: 6.2% of the values were outside the LOA (−715; −318 kJ·d^−1^) (ii) *SEE*_***lp***_: (1) Mean difference: 569 ± 868.6 kJ·d^−1^ (*p* < 0.001) (2) Linear regression: *R*^2^ = 0.717 (*p* < 0.001) (3) Bland–Altman: 6.2% of the participants were outside the LOA (−761; −377 kJ·d^−1^)	The assessment of SEE for 3 nights for the estimation of REE is a promising approach in clinical practice, but authors suggest to subtract 10% of the assessed SEE with the SWA to predict REE in normal-weight, overweight, and obese individuals

[37]	SWA (Inner View® Research Software 4.0)/right arm over the triceps muscle at the midpoint between the acromion and olecranon processes	IC, using Sensormedics Vmax-29N	REE	At least 20 min of IC + SWA (data collected during the first 5–10 min excluded for allowing the acclimatisation, experiment conducted after an overnight fast and after a resting period of 30 min)	Mean difference: −288.7 kJ·d^−1^ Bland–Altman: LOA (−63; 89 kJ·d^−1^) Pearson's correlation: *R* = 0.88 (*p* < 0.001)	Specific algorithms for obese individuals are needed

[40]	SWA/Inner View® Research Software 6.1/right upper arm over the triceps muscle	IC, using Quark CPET, COSMED	REE	30 min of IC + SWA (data collected during the first 10 min excluded for allowing the acclimatisation, experiment conducted under standardized condition, after an overnight fast)	*Women*: Mean difference: −173.2 kJ·d^−1^ (*p*=0.066) Pearson's correlation: *R* = 0.58 (*p* < 0.001) Pearson's regression analysis: *R* = −0.54 (*p* < 0.001) *Men*: Mean difference: −37.7 kJ·d^−1^ (*p*=0.782) Pearson's correlation: *R* = 0.73 (*p* < 0.001) Pearson's regression analysis: *R* = −0.11 (*p* < 0.01)	Despite the good accuracy showed by SWA, results were not more accurate than the established estimation equations. SWA does not represent a reliable alternative for measuring REE in obese subjects

*EE during physical exercises (experiments conducted in a laboratory)*						

[26]	SWA (model MF-SW, Inner View® Research Software 7.0 and 8.1)/center triceps brachii muscle, midway between the elbow and the shoulder	IC, Oxycon™ Mobile portable system (CareFusion Inc., San Diego, CA)	PAEE	1 structured routine (90 min) and 1 semistructured routine (64 min), both including a wide range of activities of sedentary/light, moderate, and vigorous intensity. Participants were instructed to consume only water for at least 3 hours prior to the test	*Structured routine*: Mean difference SWA 7.0-IC: 241.8 kJ (*p* < 0.01 in both women and men) Mean difference SWA 8.0-IC: 187.4 kJ (*p* < 0.01 in both women and men) ICC SWA 7.0-IC: 0.89 ICC SWA 8.0-IC: 0.89 Bland–Altman: no proportional bias for both SWA versions *Semistructured routine*: Mean difference SWA 7.0-IC: 206.7 kJ (*p* < 0.001) Mean difference SWA 8.0-IC: 51.9 kJ (*p*=0.35) ICC SWA 7.0-IC: 0.66 ICC SWA 8.0 IC: 0.90 Bland–Altman: narrower limits of agreement for SWA 8.0	Both SWA 7.0 and 8.1 overestimated EE during the structured routine when compared with IC but during the semistructured routine SWA 8.0 provided an estimate of EE not different than that of IC, showing a greater applicability in living conditions

[28]	Fitbit Charge 3-axis accelerometer (Fitbit Inc., San Francisco, California, USA)/wrist	IC, using Parvo TrueOne 2400 (Parvo Medics, East Sandy, UT, USA)	PAEE	Two walking stages at 80.5 m/min and 107.3 m/min and two running stages at 134.1 m/min and 160.9 m/min, with 5 min rest periods between each stage	*Walk at 80.5 m/min*: Overestimation of PAEE by 21.4% (*p* < 0.05) *Walk at 107.3 m/min*: Underestimation of PAEE by 11.2% (*p* < 0.05) *Jog at 134.1 m/min*: Underestimation of PAEE by 13.7% (*p* < 0.05) *Jog at 160.9 m/min*: Underestimation of PAEE by 22.5% (*p* < 0.05)	Caution should be exercised when considering caloric expenditure data from Fitbit Charge

[31]	SWA (Inner View® Research Software 6.1)/right arm over the triceps muscle at the midpoint between the acromion and olecranon processes	IC, using K4b2 COSMED	PAEE	Brief warm-up period followed by two 10 min stages of rowing at 50% (low intensity) and 70% (moderate intensity) of each subject's predetermined VO_2_ max wearing SWA + K4b2 COSMED, with a 20 min rest between each stage, in which food consumption was forbidden (at least 3 h fast and at least 3 h abstention from any physical activity before the test, SWA is worn for 15 min before data collection)	*50% VO*_*2*_ *max*: Mean difference: 38 ± 385 kJ·min^−1^ (*p* < 0.001) Bland–Altman: LOA (−11; 4 kJ·min^−1^) Pearson's correlation: *R* = 0.82 (*p* < 0.001) *70% VO*_*2*_*max*: No significant difference: (*p*=0.149) Bland–Altman: LOA (4; 5 kJ·min^−1^) Pearson's correlation: *R* = 0.87 (*p* < 0.001)	Specific algorithms to improve the accuracy of SWA to estimate EE at various intensities are needed

[33]	ActiGraph GT3X+ 3-axis accelerometer (ActiGraph, Pensacola, Florida, USA)/waist	IC, using K4b2 COSMED	PAEE	80 min, semistructured activity protocol, performing ≥12 activities from a list of 21 choices, including sedentary activities, household activities, and ambulatory and cycling activities	Underestimation of PAEE by 26%	ActiGraph underestimations of PAEE were most likely driven by the periods of time during the protocol in which the subjects performed household activities

[34]	SWA (Inner View® Research Software 6.1)/rear part of the right arm	IC, using Vmax Spectra (Sensormedics)	PAEE	Walking protocol: Session 1, velocity = 3 km/h, 0% incline; session 2, velocity = 4 km/h, 0% incline; and session 3, velocity = 5 km/h, 5% incline	*3 km/h, 0% incline*: Mean deviations from IC: 81.19 ± 23.81% Pearson's correlation: *R* = 0.79 (*p* < 0.01) *4 km/h, 0% incline*: Mean deviations from IC: 78.18% ± 33.96% Pearson's correlation: *R* = 0.63 (*p* < 0.01) *5 km/h, 5% incline*: Mean deviations from IC −7.88% ± 16.07% Pearson's correlation: *R* = 0.74 (*p* < 0.001)	Good accuracy of SWA for measuring EE in individuals with diabetes during walking under controlled conditions, probably obtained thanks to the reasonable duration of the protocol (not too short)

[9]	RT3 triaxial accelerometer)/and TriTrac-R3D Research ergometer (containing 3 accelerometers)/waist line above each hip	IC, using Sensormedics Vmax-29N	PAEE (calculated as the difference between pre-exercise EE and exercise EE). EERT3 and EE TriTrac-R3D = calculated using Weir's equation	5 min walking at 2, 3, and 4 km/h, successively, at 4% grade on a motorized treadmill, wearing RT3 and the TriTrac-R3D + Sensormedics Vmax-29N (2 minutes of warming up before initiation of each walking condition, each walking condition separated by a 10 sec period at an intermediate speed). Pre-exercise EE was determined with the subject seated in a comfortable thermal environment, 2 hours after lunch	Data available for 8 subjects *RT3*: Overestimation of PAEE by 30.6% ± 45.5% (*p* < 0.05) Bland–Altman: LOA (−117; 75 kJ/min) *TriTrac-R3D*: Overestimation of PAEE by 54.9.0% ± 65.0% (*p* < 0.05) Bland–Altman: LOA (−151; 75 kJ/min)	Mean PAEE did not differ significantly between methods within the range of walking speeds tested. However, there was a trend toward overestimation of PAEE by the TriTrac-R3D

[36]	SWA (Inner View® Research Software 4.0)/Triceps muscle	IC, using Sensormedics Vmax-29N	PAEE	5 min pedalling on a cycle ergometer at 60 rpm at a fixed load of 60 watts, 5 min stain stepping on a 16 cm bench, 5 min walking on a motorized treadmill at 3 km/h, wearing SWA + Sensormedics Vmax-29N (experiments conducted 2 h after lunch)	*Cycle ergometer*: Mean difference: 3.8 ± 6.5 kJ·min^−1^ ICC: 0.18 (95% CI = −0.20; 0.53) *Stepping*: Mean difference: 7.1 ± 7.5 kJ·min^−1^ ICC: 0.06 (95% CI = −0.32; 0.43) *Treadmill*: Mean difference: 7.6 ± 8.8 kJ·min^−1^ ICC: 0.18 (95% CI = −0.40; 0.45) Bland–Altman: no agreement for all the three activities	Specific algorithms for obese individuals are needed

[37]	Kenz Lifecorder EX accelerometer (LC; Suzuken Co. Ltd., Nagoya, Japan)/midline of the right thigh on a belt at the level of the waist	IC, by using Parvo Medics TrueOne 2400, Sandy, UT	PAEE	Six 5-minute stages of walking on a treadmill starting at 1.5 mph and increasing to 2.0 mph, 2.5 mph, 3.0 mph, 3.5 mph, and 4.0 mph while grade was constant at 0% for the duration of the test	Mean difference in all the samples 1.5 mph: 32.6 kJ·min^−1^ (*p* < 0.01) 2.0 mph: 15.5 kJ·min^−1^ (*p* < 0.01) 3.0 mph: 13.4 ·min^−1^ (*p* < 0.01) 3.5 mph: 18.0 kJ·min^−1^ (*p* > 0.05) 4 mph: 12.6 kJ·min^−1^ (*p* < 0.05) *Overweight BMI*: Significant underestimation at speeds of 1.5 mph (*p* < 0.001), 2.0 mph (*p*=0.014), 2.5 mph (*p* < 0.001), 3.0 mph (*p*=0.03), and 4.0 mph (*p*=0.007) *Obese BMI*: Significantly underestimation at all speeds except 3.0 mph (1.5 mph (*p* < 0.001), 2.0 mph (*p* < 0.001), 2.5 mph (*p* < 0.001), 3.5 mph (*p*=0.032), and 4.0 mph (*p*=0.010))	The device does not offer the accuracy needed to provide precise feedback on EE for individuals with varying BMI levels

[38]	SWA Mini (Inner View® Research) Software 7.0)/upper arm	IC, using Parvo Medics TrueOne® 2400 Metabolic Measurement System	PAEE	Two 5 d experimental conditions separated by a minimum 7 d washout: SIT condition for 8 h/d (seated) or STAND-SIT condition for 8 h/d (alternating between a standing and seated work posture every 30 min). Experiments conducted at a controlled temperature, after a fasted state ≥10 h and after abstention from alcohol, caffeine, and moderate and vigorous physical activities for 24 h before the test	Data available for 14 subjects *STAND*: Mean difference: −0.4 ± 0.7 kJ·min^−1^ (*p*=0.61) Pearson's correlation: *R* = 0.73 (*p* < 0.001) Bland–Altman: LOA (−1.10; 1.28 kJ·min^−1^) *SIT*: Mean difference: 0.5 ± 0.7 kJ·min^−1^ (*p*=0.005) *Pearson's correlation*: *R* = 0.82 (*p*=0.003) Bland–Altman: LOA (−1.40; 0.51 kJ·min^−1^)	SWA can provide reasonable EE estimates during STAND, while PAEE during SIT showed a modest overestimation. Further modifications to the SWA mini algorithms are required to improve the accuracy of EE

*TEE and PAEE in free-living conditions*						

[27]	Actical physical activity monitor omnidirectional accelerometer (Philips Respironics, Inc., Bend, Ore., USA)/waist or wrist SWA/upper part of the dominant arm IDEEA accelerometer (MiniSun LLC, Fresno, Calif., USA, five biaxial accelerometer nodes)/5 sensors that are placed on the body: 1 on the chest, 2 on the front of the thighs, and 2 on the feet	DLW	TEE and AEE	TEE measured by DLW for 2 weeks and by accelerometers for 1 week (only the week that corresponded with wearing the activity monitors was used for analysis). AEE_DLW_ was calculated as TEE − (RMR + (0.1 × TEE)), where IC was measured with Deltatrac II metabolic car	(i) *Actical waist AEE*: (1) Mean difference: −466.01 kJ/d^−1^ (*p*=0.01) (2) Bland–Altman: *R*^2^ = 0.197 (*p*=0.00) (ii) *Actical wrist AEE*L: (1) Mean difference: 813.9 kJ/d^−1^ (*p*=0.00) (2) Bland–Altman: *R*^2^ = 0.007 (*p*=0.49) (iii) *SWA TEE*: (1) Mean difference: −251 kJ/d^−1^ (*p*=0.44) (2) Bland–Altman: *R*^2^ = 0.054 (*p*=0.22) (iii) *SWA AEE*: (1) Mean difference: −1745 kJ/d^−1^ (*p*=0.00) (2) Bland–Altman: *R*^2^ = 0.286 (*p*=0.00) (iv) *IDEEA TEE*: (1) Mean difference: 509.2 kJ/d^−1^ (*p*=0.17) (2) Bland–Altman: *R*^2^ = 0.035 (*p*=0.33) (v) *IDEEA AEE*: (1) Mean difference: 454.8 kJ/d^−1^ (*p*=0.21) (2) Bland–Altman: *R*^2^ = 0.080 (*p*=0.15)	The performance of the Actical was poor, while the IDEEA accurately estimated AEE when compared with DLW, and both the Sensewear and the IDEEA produced relatively accurate estimates of TEE

[32]	Caltrac accelerometer	DLW	TEE (kJ·d^−1^) TEE_Caltrac_ = calculated using an equation that included weight, height, age, gender, and body accelerations	TEE measured by DLW for 2 weeks accelerometer on days 1, 3, 6, 8, and 12, during the time the subjects were awake	*Mean difference*: 77 kJ·d^−1^ *Pearson's regression*: R = 0.33 (*p*=0.15) *Bland–Altman*: large individual differences	Equations used for predicting EE from accelerometer are inaccurate at individual level

[35]	SWA (Inner View® Research Software 6.1 and 5.1)/over the right triceps muscle	DLW	TEE_DLW_ (kJ·d^−1^) and AEE_DLW_ (calculated as 0.9 TEE − RMR measured by IC on a Deltatrac II respiratory gas analyser); TEE_SWA_ (kJ·d^−1^) and AEE_SWA_ (calculated as 0.9 TEE − RMR estimated using the Harris–Benedict equation)	TEE and AEE measured by DLW and SWA for 2 weeks	*TEE SWA 6.1*: Mean difference: 117 ± 941 kJ·d^−1^ ICC*:* 0.896 Pearson's correlation: *R* = 0.893 (*p* < 0.001) Bland–Altman: all values except one with LOA of 1882 kJ ***TEE SWA 5.1***: Mean difference: 105 ± 883 kJ d^−1^ *ICC*: 0.904 Pearson's correlation: *R* = 0.901 (*p* < 0.001) Bland–Altman: all values within LOA of 1766 kJ *AEE SWA 6.1* Mean difference: −653 ± 828 kJ·d^−1^ ICC: 0.643 Pearson's correlation: *R* = 0.760 (*p* < 0.001) Bland–Altman: all values except one within LOA of 1657 kJ *AEE SWA 5.1*: Mean difference: −452 ± 774 kJ·d^−1^ ICC: 0.720 Pearson's correlation: *R* = 0.786 (*p* < 0.001) Bland–Altman: all values except two within LOA of 1548 kJ	Measures of TEE from DLW and the SWA were strongly correlated and demonstrated strong agreement, and the Bland–Altman analysis revealed no systematic bias. SWA underestimated AEE but measures of AEE from DLW and SWA were strongly correlated and demonstrated moderate agreement, and the Bland–Altman analysis revealed no systematic bias

[9]	RT3 triaxial accelerometer and TriTrac-R3D research ergometer (containing 3 accelerometers)/waist line above each hip	DLW	PAEE (PAEE_DLW_ = TEE × 0.9 −RMR_IC_, PAEE_RT3_ and PAEE _TriTrac-R3D_ = PAEE/predicted RMR × RMR_IC_)	PAEE measured for 2 weeks	RT3 Mean difference: −284.5 kJ·d^−1^ Pearson's correlation*: R* = 0.67 (*p* < 0.05) Bland–Altman: LOA (−1610,607 kJ·d^−1^) TriTrac-R3D: Data available for 12 subjects Mean difference: −401.7 kJ·d^−1^ Pearson's correlation: *R* = 0.36 (*p*=0.25) Bland–Altman: LOA (−3711; 2469 kJ·d^−1^)	RT3 provides more accurate estimations than TriTrac-R3D. RT3 is a valuable instrument for the evaluation of AEE at group level, but there is a need to compensate for the underestimation in free-living conditions. At individual level, both the accelerometers present some limitations

[39]	TracmorD triaxial accelerometer system (Maastricht, the Netherlands)	DLW	TEE_,_ PAL calculated as TEE divided by SMR (measured by an overnight state in a respiration chamber), AEE calculated as (0.9 × TEE) −SMR, AEEkg calculated as AEE/BMI. Prediction equations for TracmorD : AEE = 24.113 × MCounts/day + 8.5231 PAL = 0.3218 × MCounts/day + 1.2766	PAL and AEE measured from TracmorD and DLW	47% of the variance of PAL and 58% of the variance of AEEkg were explained by the prediction equations *Bias* AEEkg = 17%; PAL = −1%	Two published equations derived with TracmorD allow valid assessment of physical activity in overweight and obese subjects

AEE: activity energy expenditure; DLW: doubly labelled water; EE: energy expenditure; EP: early uninterrupted phase of sleep; IC: indirect calorimetry; LP = late uninterrupted phase of sleep; PA: physical activity; PAL: physical activity level; REE: resting energy expenditure; RMR: resting metabolic rate; SEE: sleep energy expenditure; SIT: sitting; SMR: sleeping metabolic rate; STAND: standing; SWA: SenseWear Armband; TEE: total energy expenditure; VO_2_ max: maximal oxygen uptake.

## Abbreviations

AEE: Activity EE
BMI: Body mass index
DLW: Doubly labelled water
EE: Energy expenditure
MAPE: Mean absolute percentage error
PA: Physical activity
PAEE: Physical activity energy expenditure
QUADAS-2: Quality assessment of diagnostic accuracy studies
REE: Resting energy expenditure
RMR: Resting metabolic rate
SEE: Sleep energy expenditure
SWA: SenseWear Armband
TEE: Total energy expenditure
VO_2_ max: Maximal oxygen uptake.

## Supplementary Materials

Supplementary Materials Figure S1: Pearson's correlation/regression between accelerometer and indirect calorimetry estimates of resting energy expenditure (REE). Each horizontal bar represents the coefficient (*R* value). Figure S2: Pearson's correlation/regression between accelerometer and indirect calorimetry estimates of energy expenditure during different physical exercises in laboratory conditions. Each horizontal bar represents the coefficient (*R* value). Figure S3: Pearson's correlation/regression between the accelerometer and doubly labelled water estimates of energy expenditure under free-living conditions (AEE = activity energy expenditure; TEE = total energy expenditure). Each horizontal bar represents the coefficient (*R* value). Figure S4: mean difference between accelerometer and indirect calorimetry (IC) estimates of resting energy expenditure (REE) (expressed in kJ/day). Figure S5: mean difference between accelerometer and indirect calorimetry estimates of energy expenditure during different physical exercises under laboratory conditions (expressed in kJ/min). Figure S6: mean difference between accelerometer and the doubly labelled water estimates of the activity energy expenditure (AEE) under free-living conditions (expressed in kJ/day). Figure S7: mean difference between accelerometer and doubly labelled water estimates of the total energy expenditure (TEE) under free-living conditions (expressed in kJ/day).
