# Supplementary material for: Validity of Accelerometers for the Evaluation of Energy Expenditure in Obese and Overweight Individuals: A Systematic Review
Source: J Nutr Metab. 2020 Aug 4;2020:2327017. doi: 10.1155/2020/2327017 (PMC7424495; doi:10.1155/2020/2327017)
Supplement: Supplementary Materials — Figure S1: Pearson's correlation/regression between accelerometer and indirect calorimetry estimates of resting energy expenditure (REE). Each horizontal bar represents the coefficient (R value). Figure S2: Pearson's correlation/regression between accelerometer and indirect calorimetry estimates of energy expenditure during different physical exercises in laboratory conditions. Each horizontal bar represents the coefficient (R value). Figure S3: Pearson's correlation/regression between the accelerometer and doubly labelled water estimates of energy expenditure under free-living conditions (AEE = activity energy expenditure; TEE = total energy expenditure). Each horizontal bar represents the coefficient (R value). Figure S4: mean difference between accelerometer and indirect calorimetry (IC) estimates of resting energy expenditure (REE) (expressed in kJ/day). Figure S5: mean difference between accelerometer and indirect calorimetry estimates of energy expenditure during different physical exercises under laboratory conditions (expressed in kJ/min). Figure S6: mean difference between accelerometer and the doubly labelled water estimates of the activity energy expenditure (AEE) under free-living conditions (expressed in kJ/day). Figure S7: mean difference between accelerometer and doubly labelled water estimates of the total energy expenditure (TEE) under free-living conditions (expressed in kJ/day). [file 2327017.f1.docx]

**Supplementary material 1: Mean difference and Pearson’s correlation/regression coefficients**

Studies are included in the plots based on the availability of the corresponding statistical parameters. For each study, the validated accelerometer is reported. In the case of the SenseWear Armband (SWA), the version of the software used in the analysis is also indicated.

**Pearson's correlation/regression coefficients**


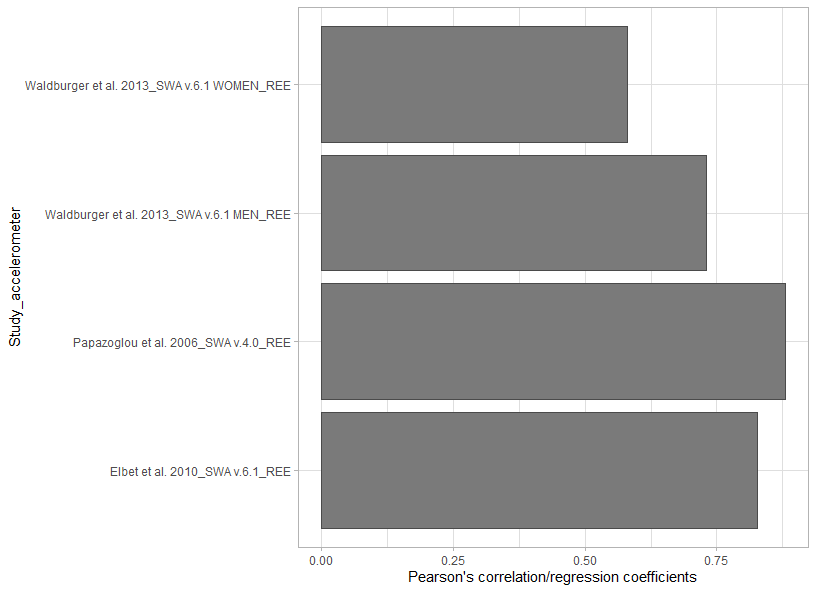


**Figure S1.** Pearson’s correlation/regression between accelerometer and indirect calorimetry estimates of Resting Energy Expenditure (REE). Each horizontal bar represents the coefficient (r value).


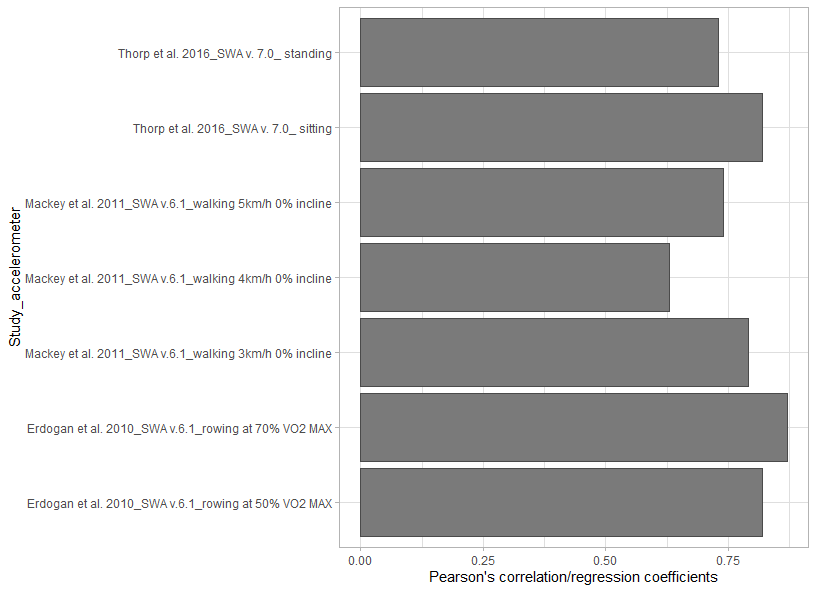


**Figure S2.** Pearson’s correlation/regression between accelerometer and indirect calorimetry estimates of Energy Expenditure during different physical exercises in laboratory conditions. Each horizontal bar represents the coefficient (r value).


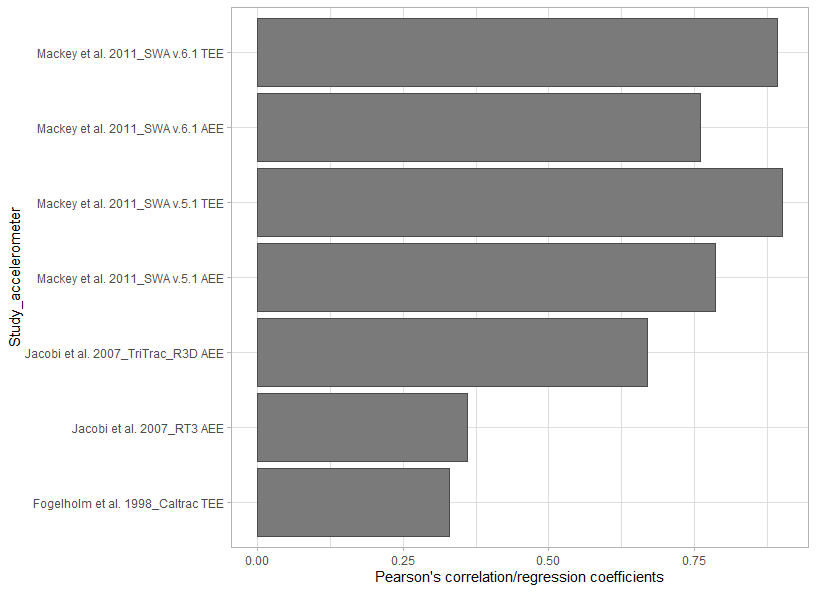


**Figure S3.** Pearson’s correlation/regression between the accelerometer and doubly labelled water estimates of Energy Expenditure under free-living conditions (AEE= Activity Energy Expenditure, TEE= Total Energy Expenditure. Each horizontal bar represents the coefficient (r value).

**Mean difference**


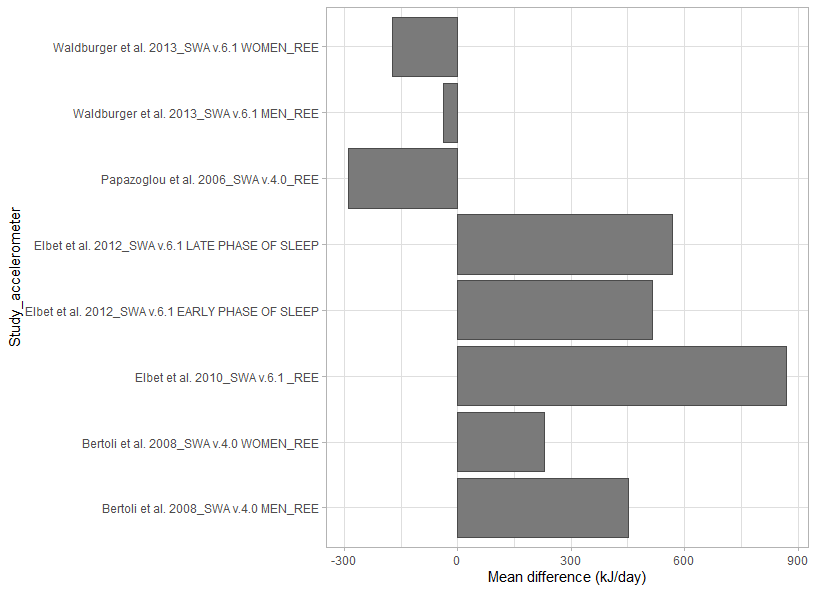


**Figure S4.** Mean difference between accelerometer and indirect calorimetry (IC) estimates of Resting energy expenditure (REE) (expressed in kJ/day)


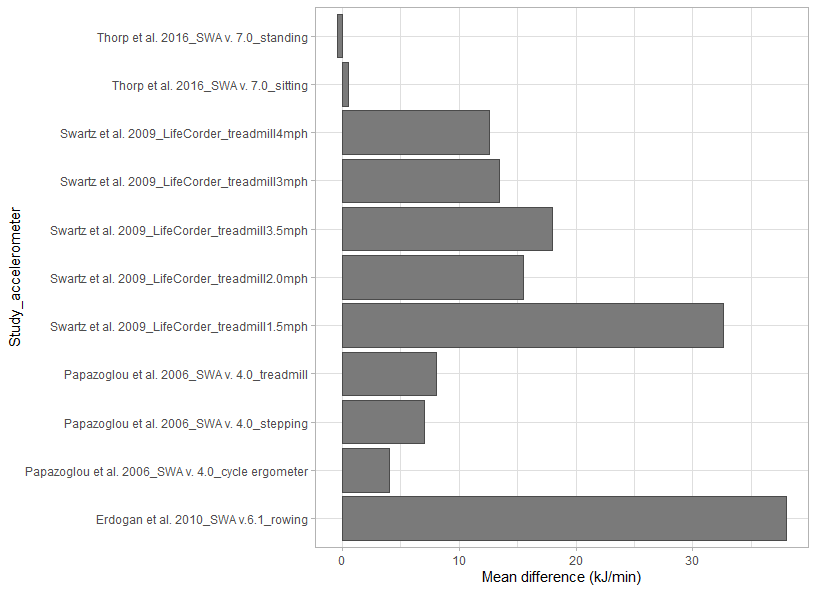


**Figure S5**. Mean difference between accelerometer and indirect calorimetry estimates of Energy Expenditure during different physical exercises under laboratory conditions (expressed in kJ/min)


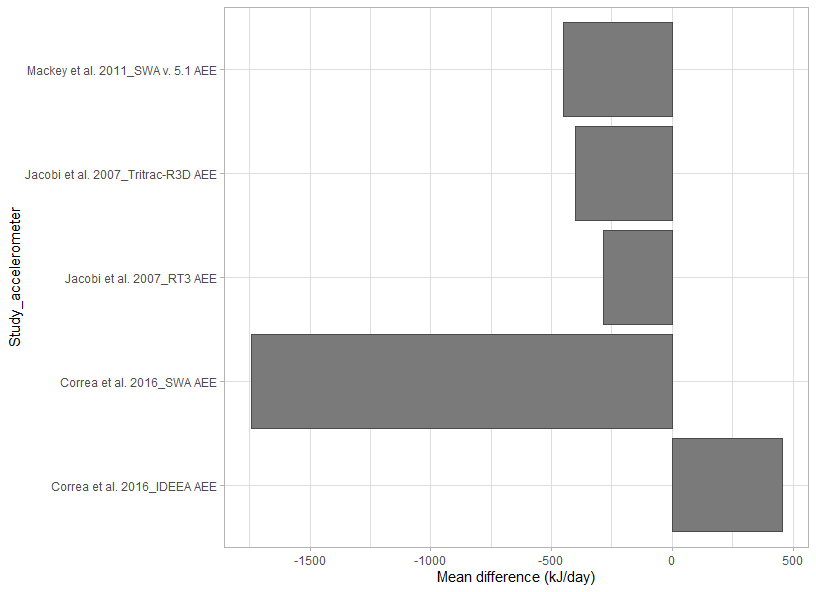


**Figure S6.** Mean difference between accelerometer and the doubly labelled water estimates of the Activity Energy Expenditure (AEE) under free-living conditions (expressed in kJ/day)


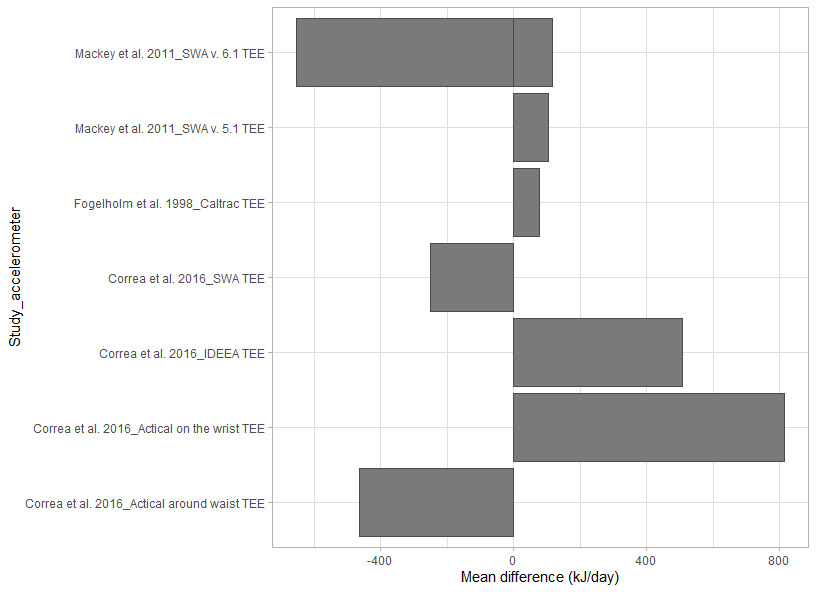


**Figure S7**. Mean difference between accelerometer and doubly labelled water estimates of the Total Energy Expenditure (TEE) under free-living conditions (expressed in kJ/day)
